# Making and Closing the Incision in Abdominal Surgery

**Published:** 1894-06

**Authors:** J. B. S. Holmes

**Affiliations:** Atlanta, Ga.; President Tri-State Medical Association, Vice-President Southern Surgical and Gynecological Association, Ex-President Georgia State Medical Association, Fellow American Association of Obstetricians and Gynecologists, Member American Medical Association


### MAKING AND CLOSING THE INCISION IN ABDOMINAL SURGERY.*

By J. B. S. HOLMES, M. D.,

ATLANTA, GA.,

President Tri-State Medical Association, Vice-President Southern Surgical and Gynecological Association, Ex-President Georgia State Medical Association, Fellow American Association of Obstetricians and Gynecologists, Member American Medical Association.

I hope I may be pardoned for taking the time of this Association by bringing up a subject upon which so much has been written and said. Many surgeons, in doing abdominal work, pay more attention to time than the details that are necessary to secure results that are free from sequelæ. This not only reflects seriously upon the surgeon, but leaves the patient, in many instances, a sufferer for life, requiring often a second operation to cure what might have been prevented.

A smooth wound, with no cicatricial tissue, no abscess scars and no ventral hernia, is a pleasing sight, not only to the surgeon, but the patient. Notwithstanding the scar or hernia is unseen by the public, the consciousness to the patient, especially if a woman, that it exists, to say nothing of the inconvenience, discomfort and pain

*Read before the Georgia State Medical Association at Atlanta, Ga., April, 1894.

from it, is always a source of mortification and sorrow. They place more stress upon these than we realize. A surgeon, like the mechanic, is, to a great extent, judged by the appearance of his work. How soon will short, deformed and crooked limbs, following fracture, destroy the reputation of the general surgeon. Then, why will not stitch hole abscess, painful indurations, ugly and tender cicatrices and ventral hernia react upon and damage the professional skill and reputation of the abdominal surgeon? The prevention of these sequelæ is my excuse for trespassing, if it prove to be such, upon your time. I do not, of course, pretend to say that they can always be prevented, but certainly with proper care and attention to the technique of the operation, if not prevented entirely, they can be reduced to a minimum. In pus cases, the abdominal walls, despite every precaution, may become infected, causing suppuration, followed by the unpleasant results already alluded to. The prevention of these sequelæ begins with the surgeon's hands, instruments, and in fact everything connected with his paraphernalia. Simply washing his hands with soap and water will not answer. The hands and arms should be thoroughly scrubbed with a brush and green soap and alcohol, repeating this several times, paying especial attention to the nails, a neglect of which, however perfect the balance of the detail may be, will result in disappointment in failing to secure ideal surgery. After this thorough scrubbing, wash well in a hot solution of bichloride, 1 to 1000, then in hot sterilized water, which I always use, not only jnst before beginning my surgical work, but also to immerse my hands in during the operation, as I never put a chemical of any character, however weak, into the abdomen. All instruments and towels should be thoroughly boiled. Sponges, pads, sutures, etc., must be known to be in an aseptic condition. The proper cleansing of the abdomen is too often overlooked. I think here is the source of much disappointment in doing ideal work. The skin, with its layers of epithelial scales, serves as a hiding place for debris and bacteria. How easily can they be carried by our hands and needles into the tissue and peritoneal cavity. After a thorough bath all over, liquify the fatty deposits in the skin by rubbing well with oil of turpentine, then scrub well with tincture of green soap, then remove remaining fat with ether, after which wash with solution of bichloride, 1 to 1000, then cover the abdomen with a wet bichloride towel, 1 to 1000, except in the line of the proposed incision. In cleansing the abdomen, of course the greatest care should be bestowed upon the umbilicus. If special attention is not paid to this, septic material will be sure to be left in it. In cleansing the abdomen, I never leave it to an assistant or nurse, unless under my own supervision, for a neglect to do it well is sure to bring disappointment. In making the incision use a sharp knife, making the cut with one sweep of the knife, from one end of the wound to the other, continuing this till the peritoneum is reached. Clipping a little tissue with the scissors, raising it with the grooved director, and cutting with knife or scissors, tearing with the fingers or handle of your knife is to be condemned, as it leaves rough, jagged edges that will fill with small blood clots, prevent perfect coaptation and consequently we fail to get primary union, to secure which a smooth, even surface is essential. Control all hemorrhage before the peritoneal cavity is opened. Do this, if possible, by hot sponges, avoiding if you can all ligatures and compression forceps. When ligatures are necessary, use the finest silk and cut them short. If the pressure forceps are required, two or three minutes is sufficiently long to leave them on to completely stop the hemorrhage. Longer pressure frequently results in tissue necrosis and mural abscesses. Especially is this apt to occur where pressure is used in the fatty tissue, for we must remember that this is little else than oil globules in loose connective tissue, poorly supplied with nutrient blood vessels and consequently of low vitality. Make your incision, when possible, directly in the linea alba.

You then have no muscles to cut, no fascia in front or behind the muscle to divide with its consequent uneven and irregular retraction, which makes it almost impossible to get satisfactory apposition. Unfortunately, many good surgeons place too little stress on the linea alba. The strength and support of the abdomen is not from the muscles but from the fascia. However well the muscles unite, if the fascia does not, we are sure to have hernia. The linea alba, as you know, is the aponeurosis of the oblique and transversallis muscles, with no muscular tissue in it. If the incision is made in the median line, you should have no difficulty in finding it. When the peritoneum is reached, raise it with the catch forceps and clip it with a scalpel. Insert your finger in the abdomen as a guide, then slide knee bent scissors upon it, enlarging the opening in one or both directions, as you like. By slipping the finger along the peritoneal wall of the abdomen, as a guide, it is impossible to do damage with your scissors to the viscera. As to the length of the incision, there is still much dispute, both as to the mortality due to, and the post operative sequelæ attributable to it. Long incisions are to be avoided, when possible, for numerous reasons. The increased exposure of the intestines and peritoneum to the air, the constant manipulation necessary to keep the intestines in the abdomen, the prolonged anesthesia required for closing the wound, all add greatly to the shock. Ordinarily, an incision, for the removal of ovaries and tubes that will admit two fingers is sufficiently long. The educated surgical finger will break up adhesions without seeing the viscera. Universally adherent or solid tumors, or adhesions to the small intestines, require larger incisions and occular inspection may be necessary. We should, however, in all cases make the incision sufficiently large to do the work with ease, for in attempting to work through a small opening the peritoneum and other tissues may be bruised and seriously damaged. The first step in closing the incision is to be assured that all hemorrhage is stopped; then thoroughly cleanse the edges of the wound with hot sterilized water, removing all blood clots.

In the manner of closing and the material used there is still a great difference of opinion among many able operators. Too much surgery is bad; therefore, the simplest suture that will accomplish the best results is to be preferred. I use exclusively silkworm gut as a suture and thus far have used only the through and through method. I have had no occasion to regret this course. In some fat subjects, or where the incision had to be made through muscles, with the consequent uneven retraction of the fascia, I might use the several layers of buried suture and in this event would use a very fine silk or the kangaroo tendon. The time required for absorbing silkworm gut is too long and the catgut I regard as not reliable. I have had no expererience with the buried suture in abdominal work, but my results with it in perineal work have not been satisfactory. I can see no advantage whatever to be gained by suturing each layer of tissue separately, except in the class of cases already referred to. The operation is sure to be prolonged and a lot of slow absorbing material is left in the tissue that may set up irritation and prove troublesome to remove. Abscesses often form as a result of this irritation and hernia frequently follows. I use a long, slightly curved needle without a cutting surface. Needles with cutting surfaces frequently cause hemorrhage in the abdominal wall and abscesses result. With the index finger of my left hand in the abdomen and the thumb on the skin, the peritoneum and skin are slightly retracted. The needle, armed with silkworm gut, is then passed through the skin about one-quarter of an inch from the margin and with the concave side looking toward the incision is passed through all the tissues and is brought out through the peritoneum one-eighth of an inch from the margin. If the abdominal walls are thin, then the needle is carried through the tissues on the oppoite side before withdrawing it. After all the stitches are passed the ends are brought together and some traction made. It will be seen that the peritoneum fascia and all intervening tissues are brought together before the skin is. You will see, while we have only a little peritoneum and skin included in the suture, we have quite an inch of intervening tissue, and as the traction of the suture is almost entirely on the fascia and muscle, we are sure its apposing edges are held together. A good union results and the strength of the abdominal wall is maintained. Before the stitches are tightened we must see that all the tissues of like character are in apposition. This must not be overlooked or neglected; otherwise a weak wall is the result and hernia will likely follow. All the ends of the sutures are brought together and gentle traction made. If we are sure that the tissues are in proper apposition, the sutures are held by an assistant, and I now tie, usually beginning with the lowest and working upward. When the first stitch is tied the knot is on the skin, resting against the second stitch; as the suture is tightened, it is allowed to glide downward on the skin till it reaches its proper place. Each suture is then treated in this way till the wound is closed. If you have never tried this gliding suture, you will be surprised to see how beautifully the edges of the skin are brought in apposition. Should the skin on the entire line of the incision not be brought perfectly together, a few superficial sutures of the finest silk may be inserted. Do not draw your sutures too tight, not enough to pucker the skin, or they will give great pain, compress the subcutaneous fat and produce mural and stitch-hole abscesses. I allow my sutures to remain from six to nine days. If the patients present no symptoms to cause me to suspect wound irritation, the dressings are undisturbed until the sixth or seventh day, when every alternate deep and all superficial sutures are removed; two days later the others are removed. If there is any temperature after the third day, I remove the dressings and examine to see if there is any suture irritation. If so, I remove a part of them, leaving enough, however, to give support. The incision, after the sutures are applied, is dressed with iodoform; then a pad or compress of sterilized gauze is placed over this and then bichloride cotton. I then apply across the abdomen, but not too tight, three or four strips of adhesive plaster about two inches in width. These are fastened to the sides of the patient, and are intended to take the tension and strain from the stitches in case of severe vomiting or great distention from gas. Finally a soft flannel binder is applied and the patient put to bed.

I again ask your pardon for bringing up a subject so old, but nothing pays so well as the little details in our surgical work, and especially is this true in caring for the incision in abdominal work. It not only gives good results so far as the future of the patient is concerned, but when she sees that her wound is healed without suppuration a fine effect upon her is produced. If there is even a stitch-hole abscess that requires care and attention, my experience has been that it has quite a depressing effect upon the patient, despite all your efforts at explanation. She feels that in a measure your operation has been unsuccessful.

